# rRNA-gene methylation and biological aging

**DOI:** 10.18632/aging.101369

**Published:** 2018-01-24

**Authors:** Patrizia D’Aquila, Dina Bellizzi, Giuseppe Passarino

**Affiliations:** 1 Department of Biology, Ecology and Earth Science, University of Calabria, 87036, Rende, Italy

**Keywords:** aging, biological age, chronological age, ribosomal DNA (rDNA) promoter, ribosomal RNA (rRNA) genes

Epigenetic modifications, which are programmed across the individual’s lifespan by the genetic background but also modulated by environmental and lifestyle factors, are widely studied to identify biomarkers of human aging. Indeed, Garagnani et al. identified three genes whose methylation level strongly correlates with age [1]. Subsequently, Horvarth defined a high precise DNA methylation clock, able to predict age, with a low margin of error, based on methylation levels of a series of CpG sites scattered throughout the genome. These markers, and others which have consequently been identified, have proved to be reliable enough to be used for forensic purposes in order to define the age of human subjects by analysing these markers in different specimens (such as teeth, blood, semen) [2].

On the other hand, most of the scientists involved with research on aging are willing to identify epigenetic modifications correlated with biological rather than with chronological age. Such markers may be important firstly because the definition of biological age in the elderly may be much more useful than chronological age to identify the subjects needing (medical or social) care; secondly, because the identification of genes the methylation of which is correlated with biological aging may give important clues on the aging process and, possibly, on how to modulate it. Many hints suggest that these markers are likely to exist. For instance, global methylation showed to be correlated with biological and not with chronological age [3].

Ribosomal RNA (rRNA) genes consist of highly evolutionary conserved tandem and clusters of units within nucleolar organizer regions, distributed, in humans, on the short arms of acrocentric chromosomes. The expression of rRNA genes is regulated during different stages of lifetime by several factors, including nutrients, transcription factors and epigenetic marks as well [4,5]. Previous studies have indicated that alterations of rRNA expression may be related to specific pathological conditions, such as different types of cancers and Alzheimer Disease [6]. In addition, the analysis of fibroblasts from patients with Werner Syndrome, and of hematopoietic cells from rats of different ages suggested a correlation with physiological and pathological aging. These data, coupled with the acknowledged importance of protein use and storage in the elderly, attracted our interest, suggesting that the expression of rRNA genes may be correlated to the functional status of old subjects and, consequently, that the evaluation of the epigenetic status of rRNA could be informative regarding the quality and the rate of the individual aging process [7].

We evaluated the methylation levels of 19 CpGs located within the upstream control element (UCE) and 8 CpGs in the core promoter of rDNA in three groups of unrelated humans, each including subjects of different ages. For each subject the data regarding the cognitive, functional, and psychological status were collected and analysed. In addition, a follow up study allowed us to consider the vital status after nine years. Although a slight association between the methylation levels of some of the CpGs analysed and chronological age was observed, the methylation status of rRNA promoter appeared highly correlated with the cognitive performance and the survival chance. In fact, a significant negative correlation between the methylation levels of a specific CpG site (CpG_5) and the results of two test of episodic memory, that consist in the immediate and delayed recall of a 12-word list (r=-0.128; P=0.036), was observed. In addition, subjects of 60-89 years old and displaying higher methylation levels at CpG_5 have a mortality risk increased of about two fold with respect to age-matched subjects with low methylation levels in a 9-year follow-up period (HR=2.073; P=0.002). The results were consistently replicated in the three independent population samples, making these results quite reliable.

Being the rDNA a highly evolutionary conserved region, we extended the characterization of its methylation levels to the homologous region of rats by analysing 9 CpGs in DNA samples extracted from blood, heart, liver, kidney, and testis of 3, 28, 40, 88, and 96 weeks old. A linear regression analysis of the methylation levels as a function of age (which, in rats of the same strain raised in the same conditions reflects biological age much better than in humans) revealed that, regardless of the tissue type, the methylation of the rRNA promoter region exhibits a progressive and general increase, with the most important variation occurring in blood, where an about tenfold increase has been observed from weaning to old age.

The analysis of rRNA in tissues of both humans and rats with different levels of methylation, confirmed the inverse correlation between the methylation and the transcription levels of rDNA and, then, the suppressive role of DNA methylation in silencing gene expression.

These results, provide an interesting biomarker of the biological aging but they are also useful to understanding one of the most important age related process, that is the (in some cases dramatic) decline in the overall protein synthesis, which may have important effects, such as sarcopenia. If this decline has so far being justified as the result of a reduced translation frequency, our findings demonstrate an epigenetically-driven negative regulation of ribosomal RNA gene expression.
